# Oour experience in the early management of patients with IgE- mediated cow’s milk allergy (CMA)

**DOI:** 10.1186/2045-7022-1-S1-P105

**Published:** 2011-08-12

**Authors:** Javier Boné, Angela Claver, Isabel Guallar

**Affiliations:** 1 Hospsital Universitario Miguel Servet, Zaragoza, Spain; 2 Hospital Universitario Miguel Servet, Pediatric Allergy, Zaragota, Spain

## Background

Strict avoidance of food allergens has been advised for sensitization prevention and management. However, recent studies suggest that is the early introduction of food allergens, rather that avoidance, which might induce tolerance.

## Methods

January 2007-December 2009: 48 infants (< 1 year old) were diagnosed in our Unit of IgE mediated CMA after a complete evaluation (anamnesis and physical examination, skin prick test and specific serum IgE levels) Subsequently, ALL patients underwent an open food challenge (OFC). The OFC was postponed only in 1 case: a 4-month old boy with previous anaphylaxis whose parents refused OFC until age one.

In the OFC, we do not look for reactions, but for a well-tolerated dose as a starting point for reaching tolerance.

## Objectives

To evaluate the effectiveness and safety of our performance.

## Results

Most patients (44/48), presented a negative OFC (36/44) or mild symptoms (2 oral allergy syndrome, 1 urticaria, 1 rhinoconjunctivitis, 4 vomiting) Subsequently, patients underwent a gradual dose increase at home. Slower increases for cases with mild/moderate symptoms were recommended. The remaining patients (4/48), presented a positive OFC (3/48 mild anaphylaxis) or persistent symptoms in home dosing (1/48 daily vomiting). An elimination diet was prescribed, followed by a second challenge 3-6 months later • it was negative in 2 patients, who continued gradual increases and a free diet at age two, and positive in 2, who began a standardized desensitization treatment.

## Comments

Our results show the possibility for early management in IgE mediated CMA. An early OFC, could be not only a diagnostic tool but also the beginning of the treatment, especially in infants: Could a diagnosed infant be a treated infant?

Mild/moderate symptoms are common but usually disappear with slower increases.

We believe that by anticipating clinical tolerance, we could change the natural history of CMA in many patients.

